# Effect of an Oral Health Education Program on Knowledge, Attitude, and Clinical Oral Health Indices in Pregnant Women in Urmia, Iran: A Nonrandomized Controlled Intervention Study

**DOI:** 10.1002/cre2.70357

**Published:** 2026-04-15

**Authors:** Seyyed Amir Seyyedi, Zohreh Dalirsani, Fatemeh Bahadori, Nava Askari, Mohadese Maleki, Mohammad Reza Ghorbani Afkhami

**Affiliations:** ^1^ Department of Oral and Maxillofacial Diseases School of Dentistry, Urmia University of Medical Sciences Urmia Iran; ^2^ Oral and Maxillofacial Diseases Research Center, Mashhad University of Medical Sciences Mashhad Iran; ^3^ Department of Obstetrics and Gynecology Urmia University of Medical Sciences Urmia Iran; ^4^ Student Research Committee, Urmia University of Medical Sciences Urmia Iran; ^5^ Research Assistant Urmia University of Medical Sciences Urmia Iran

**Keywords:** dental, dental plaque index, gingival index, health education, pregnancy

## Abstract

**Objectives:**

Hormonal changes during pregnancy can increase susceptibility to plaque‐induced gingivitis, and at the same time, dental care visits during pregnancy have been reported to be low. This study evaluated the effect of an oral health education program on knowledge, attitude, and clinical indices of oral health in pregnant women.

**Methods:**

This nonrandomized controlled intervention study was conducted at Kowsar Hospital, Urmia. Allocation to groups was based on the day of visit (even/odd days). In addition to routine prenatal care, the intervention group received an individual education session of approximately 60 min with a brochure, and the control group received routine care only. Knowledge, attitude, plaque index (PI), gingival index (GI), and decayed, missing, and filled teeth (DMFT) indices were measured at baseline and 3 months later. Statistical analysis was performed in Statistical Package for the Social Sciences.

**Results:**

Of 240 enrolled participants, 200 (100 per group) provided complete follow‐up data. Baseline characteristics and oral health indices were comparable between groups. At 3 months, the intervention group demonstrated improved knowledge and attitude, lower PI, and modest improvement in GI. In the control group, knowledge and attitude changed little, GI worsened, and PI remained largely unchanged. DMFT increased in both groups over the 3‐month period.

**Conclusions:**

Short‐term, structured oral health education with a brochure can improve knowledge and attitude of pregnant women in the short term and improve plaque and gingivitis.

**Abbreviations**
DMFT: decayed, missing, and filled teeth
GI: gingival index
PI: plaque index
SPSS: Statistical Package for the Social Sciences

## 1 Introduction

Oral health is an integral part of overall health and should be maintained throughout life, including during pregnancy (Committee Opinion No. 569 2013). Physiological and hormonal changes during pregnancy, particularly increased estrogen and progesterone levels, can exacerbate the inflammatory response of gingival tissue to microbial plaque and lead to the development of “pregnancy gingivitis,” which has been reported to occur in 60%–70% of pregnant women (Wu et al. 2015; Sachelarie et al. 2024).

Several observational studies and systematic reviews have shown that periodontal disease during pregnancy is associated with an increased risk of adverse pregnancy outcomes such as preterm birth, low birth weight, and preeclampsia (Daalderop et al. 2018; Chen et al. 2022).

Accordingly, authoritative clinical statements and guidelines, including the American College of Obstetricians and Gynecologists (ACOG) Committee Opinion, emphasize that preventive care and dental treatments during pregnancy are safe and should be considered as part of routine prenatal care (Committee Opinion No. 569 2013). In addition, routine oral health examination and periodontal status assessment during pregnancy are recommended to enable timely identification and management of gingival/periodontal conditions (Capasso et al. 2016).

Several studies have shown that pregnancy is often associated with an exacerbation of oral and dental problems (Rakchanok et al. 2010; Pecci‐Lloret et al. 2024). In a study, more than 74% of pregnant women had at least one carious tooth, and more than 86% had symptoms of gingivitis (Rakchanok et al. 2010). Recent systematic reviews have also reported that the prevalence of periodontal disease in pregnancy can range up to 46% for periodontitis and up to 30%–100% for gingivitis in some populations (Pecci‐Lloret et al. 2024).

Despite this, the use of dental services during pregnancy has been reported to be low; many pregnant women do not visit a dentist even if they have oral problems, and the most important barriers mentioned include lack of awareness of the need for periodic check‐ups, lack of feeling of need, fear of harming the fetus from treatment, financial and access problems (Subedi et al. 2024; Baskaradoss and Geevarghese 2020; Onwuka et al. 2021).

Findings in Iran also show a similar picture. In a study of pregnant women in Ilam, the level of knowledge, attitude, and practice regarding oral health was reported to be “average” (Daneshvar et al. 2023). In a study on pregnant women in Urmia, the level of knowledge and attitude toward oral health was assessed as “unfavorable” and oral health indicators, including plaque and gingival status, needed serious improvement; the authors emphasized the need for targeted educational programs for this group (Seyyedi et al. 2023).

In recent years, several intervention studies have attempted to evaluate the effectiveness of oral health education programs on knowledge, attitudes, and clinical indicators in pregnant women (Selvarajan et al. 2019). In an intervention study in India, oral health education was provided to pregnant women in the form of face‐to‐face sessions and brochures, and the results showed that the intervention resulted in significant increases in knowledge and improvements in periodontal indicators, including reduced plaque and gingivitis (Selvarajan et al. 2019; Chawla et al. 2017).

In a family‐based controlled trial in China, family‐based behavioral and educational counseling was reported to be more effective than traditional brochure‐based education in improving oral health status and periodontal indices in pregnant women (Liu et al. 2020). Also, a recent randomized trial based on the health belief model showed that an oral health management program can significantly improve self‐efficacy, oral health behaviors, and periodontal indices including plaque index (PI) and gingival index (GI) in pregnant women (Chen et al. 2025).

In Iran, several educational interventions have also been conducted on pregnant women. In the study by Mohammadkhah et al. education based on the theory of planned behavior significantly improved oral health behaviors and clinical outcomes in pregnant women, highlighting the importance of using behavioral frameworks in the design of interventions (Mohammadkhah et al. 2023). In an intervention study by Rezaei et al. on pregnant women in Mashhad, the intervention group received oral health education and after 3 months, a significant increase in knowledge, improved attitude, and a decrease in plaque index were observed in this group (Rezaei et al. 2023).

Despite this evidence, a significant portion of the available studies, especially in Iran, still have important limitations. Many studies conducted on pregnant women have been cross‐sectional or quasi‐experimental and have limitations in terms of true randomization, sample size, and medium‐ or long‐term follow‐up (Daneshvar et al. 2023; Seyyedi et al. 2023; Mohammadkhah et al. 2023; Rezaei et al. 2023). Furthermore, in a large proportion of educational interventions, the main focus has been on self‐reported indicators such as knowledge, attitude, and behavior, and a comprehensive set of clinical indicators including PI, GI, and decayed, missing, and filled teeth (DMFT) have not been assessed simultaneously and over time (Selvarajan et al. 2019; Chawla et al. 2017; Chen et al. 2025). These limitations, especially in the Iranian context, highlight the need to design and conduct a study in different populations of pregnant women to assess the effect of oral health education programs on cognitive and clinical outcomes.

Based on the results of a previous study conducted in Urmia that showed that the level of knowledge and attitude of pregnant women towards oral health and hygiene was not desirable, the present study was designed among pregnant women referring to Kowsar Hospital in Urmia to evaluate the effect of an oral health education program on the level of knowledge and attitude towards oral health, as well as on the clinical status of the mouth based on PI, GI, and DMFT over a 3‐month period.

## 2 Methods

### 2.1 Study Design

This nonrandomized controlled intervention (quasi‐experimental) study was conducted on pregnant women referred to Kowsar Hospital in Urmia from July 22, 2024, to February 18, 2025. Data collection and intervention implementation were performed by two final‐year dental students under the supervision of an oral and dental specialist and an obstetrician and gynecologist.

### 2.2 Participants

The study population was systemically healthy pregnant women who were referred for routine prenatal care. Inclusion criteria included general health, gestational age of approximately 12 weeks, minimum primary education, ability to read Persian, and informed consent. Exclusion criteria included systemic diseases affecting the gums, use of certain medications or antibiotics in the last 3 months, tobacco use, urgent need for dental treatment, major pregnancy complications, and failure to attend follow‐up.

Considering the expected attrition rate, 120 participants were included in each group (total 240 participants) and finally the complete data of 100 participants in the intervention group and 100 participants in the control group were used for analysis. Sampling was done on a convenience basis from eligible clients.

### 2.3 Allocation and Blinding

Allocation was not randomized; participants were assigned based on day of visit (even/odd days) due to logistical constraints; pregnant women visiting on even days of the week (Saturday, Monday, and Wednesday) were placed in the intervention group, and those visiting on odd days of the week (Sunday, Tuesday, and Thursday) were placed in the control group. Therefore, the study should be interpreted as nonrandomized, with a potential risk of selection bias. At the beginning, a dental student who was not aware of the allocation method administered the questionnaires and baseline examinations. Then, a second student conducted an educational session for the intervention group. Participants were asked not to talk to the examiner about receiving the education at the follow‐up visit; therefore, the examiner who recorded the clinical indicators at two points was blinded to the grouping of the samples.

### 2.4 Knowledge and Attitude Questionnaire

Demographic information and knowledge and attitude status regarding oral health were collected with a questionnaire based on the study by Rezaei et al. (2023). The questionnaire consisted of two sections: knowledge and attitude, and the scoring system is summarized in Table 1. The questions in the knowledge section generally assessed individuals’ knowledge about the importance of oral health during pregnancy, its possible effects on mother, fetus, and infant oral health, the necessity of dental examination before and during pregnancy, common misconceptions, and the appropriate timing and safety of dental care and treatments during pregnancy. The questions in the attitude section generally assessed individuals’ beliefs and perceptions about the importance of oral health during pregnancy, the necessity of visiting a dentist, perceptions of the safety or harmfulness of dental treatments and radiographs, and the tendency to postpone treatment or use unprofessional methods. The questionnaire was completed in the form of a structured interview by trained students.

**Table 1:** Scoring and classification criteria for questionnaire scores (knowledge and attitude) and clinical indices (GI and PI).

Panel A. Questionnaire score classification		
Variable	Condition	Score range
Knowledge	Very good	+7 to +10
	Good	+3 to +6
	Intermediate	−2 to +2
	Poor	−6 to −3
	Very poor	−10 to −7
Attitude	Completely positive	+11 to +16
	Positive	+4 to +10
	Neutral	−3 to +3
	Negative	−10 to −4
	Completely negative	−16 to −11

**Panel B. Clinical indices scoring criteria**		
**Index**	**Description**	**Score**
GI	Normal gingiva; no signs of inflammation	0
	Mild inflammation; slight change in color or slight edema; no bleeding on probing	1
	Moderate inflammation; redness and edema present; bleeding occurs on probing	2
	Severe inflammation; marked redness and edema, possible ulceration, tendency to bleed spontaneously	3
PI	No plaque visible	0
	Separate small flecks of plaque at the cervical margin of the tooth	1
	Thin continuous band of plaque at the cervical margin of the tooth	2
	Band of plaque wider than 1 mm but covering less than one‐third of the crown of the tooth	3
	Plaque covering at least one‐third but less than two‐thirds of the crown of the tooth	4
	Plaque covering two‐thirds or more of the crown of the tooth	5

Abbreviations: GI, gingival index; PI, plaque index.

### 2.5 Questionnaire Validity and Reliability

The questionnaire's content was assessed through expert review by two oral medicine specialists. Reliability was evaluated using a test–retest approach: the questionnaire was administered to 15 participants and re‐administered to the same participants 3 weeks later. The test–retest correlation coefficients were 0.75 for the knowledge section and 0.81 for the attitude section, indicating acceptable reliability.

### 2.6 Oral Clinical Indices

The clinical indices PI, GI, and DMFT were measured on two occasions (at the beginning of the study and 3 months later). The clinical visual intraoral examinations were performed by the same trained examiner, using a mouth mirror and Williams probe, under ambient light in the examination room. No radiographic examination was performed, and therefore the DMFT score was determined based only on clinical findings.

The scoring method for GI and PI was completely consistent with the study by Rezaei et al. (Rezaei et al. 2023). and based on the Silness and Löe criteria, and the scoring details are shown in Table 1. The DMFT index was also calculated based on the World Health Organization (WHO) criteria by recording decayed, filled, and lost teeth due to caries.

### 2.7 Educational Intervention

In addition to routine prenatal care, the intervention group received an individual oral health education session for approximately 60 min. In this manuscript, we use the term oral health education to refer to the structured educational intervention, which included oral hygiene instruction (brushing and flossing) as well as pregnancy‐related oral health guidance. The session was conducted by a final year dental student and covered topics including the importance of oral health during pregnancy, proper brushing and flossing, nutritional tips, and the safety of dental treatments during pregnancy.

At the end of the session, participants were provided with an educational brochure. The brochure included illustrated, step‐by‐step instructions on correct toothbrushing and flossing, practical recommendations for maintaining oral hygiene, and guidance on infant oral health care. Including infant oral health guidance is important because parental support and appropriately designed parental interventions can shape children's health‐related behaviors from early life (Pranno et al. 2022). The control group received only routine gynecological care and no specific structured education on oral health.

### 2.8 Timing of Measurements

For both groups, the knowledge and attitude questionnaire and PI, GI, and DMFT indices were completed and recorded at two times before the intervention and 3 months after the intervention.

### 2.9 Ethical Considerations

The study protocol was approved by the Research Ethics Committee of Urmia University of Medical Sciences (Ethics Code: IR. UMSU. REC.1398.381). Written informed consent was obtained from all participants, and they were assured that their information would be confidential and that non‐participation in the study would not affect the routine pregnancy care process.

### 2.10 Data Analysis

Data were analyzed in SPSS version 27. Quantitative variables were reported as mean and standard deviation, and qualitative variables were reported as frequency and percentage. The normality of the distribution of quantitative variables was examined with the Shapiro–Wilk test, and given the nonparametric nature of most variables, the Mann–Whitney *U* test was used to compare quantitative variables between two groups, and the Wilcoxon signed‐rank test was used for comparison before and after in each group. The relationship between knowledge and attitude scores with clinical indicators and education level was examined using the Spearman correlation coefficient. The statistical significance level in all tests was considered *p* < 0.05. Statistically significant values are indicated with an asterisk (*) in the tables.

## 3 Results

### 3.1 Baseline Characteristics of Participants

A total of 200 pregnant women (100 in the control group and 100 in the intervention group) were included in the final analysis. The mean age of the participants in the two groups was not significantly different, and other baseline indicators, including knowledge and attitude scores, as well as initial values of DMFT, PI, and GI, did not show any statistically significant differences between the control and intervention groups at the beginning of the study; therefore, the two groups were almost homogeneous in terms of baseline cognitive and clinical oral health status (Table 2).

**Table 2:** Baseline characteristics and pre‐intervention oral health measures of participants in the control and intervention groups.

Variable	Control group (*n* = 100)	Intervention group (*n* = 100)	*p* value
Age (years)	30.11 ± 5.86 (18–44)	29.86 ± 5.83 (16–44)	0.828
Indices (mean ± SD, (min–max))			
Knowledge	4.46 ± 1.95 (−1–10)	4.35 ± 1.60 (0–8)	0.655
Attitude	8.72 ± 2.53 (2–14)	8.63 ± 3.20 (2–16)	0.913
DMFT	9.42 ± 4.12 (2–19)	9.66 ± 4.10 (2–23)	0.988
PI	2.17 ± 0.62 (1.00–3.75)	2.16 ± 0.61 (1.00–3.00)	0.931
GI	1.21 ± 0.30 (0.71–2.71)	1.20 ± 0.23 (1.00–2.17)	0.935
Educational level (N (%))			
Below diploma	28 (28.0%)	40 (40.0%)	—
Diploma	45 (45.0%)	38 (38.0%)	—
University degree	27 (27.0%)	22 (22.0%)	—

*Note:* Test: Mann–Whitney *U.*

Abbreviations: DMFT, decayed, missing, and filled teeth; GI, gingival index; PI, plaque index.

### 3.2 Education Level and Its Relationship with Indicators

**Figure 1:**
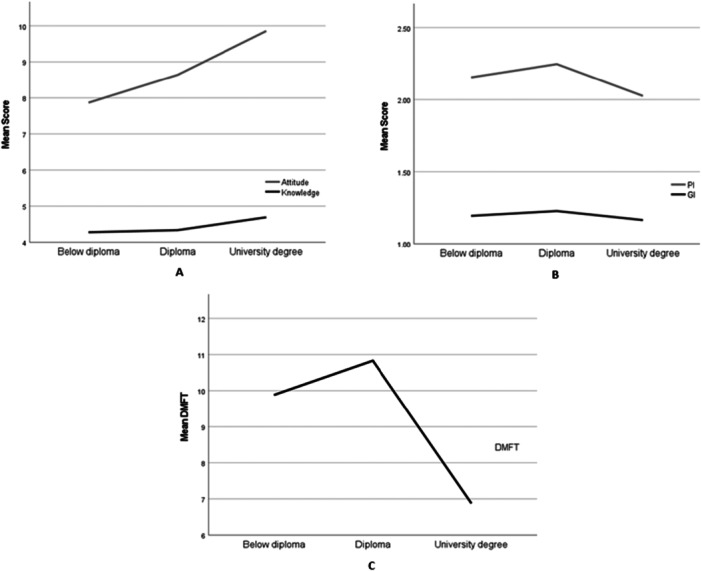
Comparison of the mean knowledge, attitude, GI, PI, and DMFT across different education levels. (A) Association between knowledge and attitude with education level. (B) Association of PI and GI with education level. (C) Association between DMFT and education level. DMFT, decayed, missing, and filled teeth; GI, gingival index; PI, plaque index.

The distribution of education level (below diploma, diploma, and university degree) in the control and intervention groups was relatively similar, although the proportion of women with below diploma education in the intervention group was slightly higher than in the control group (Table 2). To examine the relationship between education level and the study indicators, baseline (pre‐intervention) data from both groups were pooled. Comparative analysis showed that the level of education was not significantly associated with the knowledge score and PI and GI indices (*p* > 0.05), but women with university education had significantly higher attitude scores than the other two groups (*p* = 0.003) and the mean DMFT in this group was significantly lower than women with diploma and less diploma education (*p* < 0.001). These differences across education levels are summarized in Figure 1. The results indicate that higher education is associated with more positive attitude and less experience of cumulative caries, although it did not produce a clear difference in knowledge and short‐term inflammatory indices.

### 3.3 Changes in Knowledge, Attitude, and Clinical Indices in the Control Group

In the control group, knowledge and attitude scores showed slight and statistically insignificant changes during the 3‐month follow‐up; in other words, in the absence of targeted educational intervention, the cognitive status of participants in this group did not improve significantly. The GI index showed a significant increase, while the PI index remained almost unchanged (Table 3).

**Table 3:** Changes in knowledge, attitude, and clinical oral health indices from baseline to 3‐month follow‐up in the control group and intervention group.

Variable/Index	Baseline mean ± SD, (min–max)	3‐month follow‐up mean ± SD, (min–max)	*p*‐value
Control			
Knowledge	4.46 ± 1.95 (−1–10)	4.74 ± 2.26 (0–10)	0.083
Attitude	8.72 ± 2.53 (2–14)	8.80 ± 3.30 (1–15)	0.855
DMFT	9.42 ± 4.12 (2–19)	9.53 ± 4.18 (2–19)	< 0.001*
PI	2.17 ± 0.62 (1.00–3.75)	2.19 ± 0.72 (0.50–4.00)	0.635
GI	1.21 ± 0.30 (0.71–2.71)	1.28 ± 0.35 (1.00–2.92)	< 0.001*
Intervention			
Knowledge	4.35 ± 1.60 (0–8)	6.24 ± 1.73 (2–9)	< 0.001*
Attitude	8.63 ± 3.20 (2–16)	9.70 ± 3.10 (1–16)	< 0.001*
DMFT	9.66 ± 4.10 (2–23)	9.88 ± 4.04 (2–24)	< 0.001*
PI	2.16 ± 0.61 (1.00–3.00)	1.83 ± 0.62 (0.50–3.25)	< 0.001*
GI	1.20 ± 0.23 (1.00–2.17)	1.16 ± 0.19 (0.83–2.04)	0.023*

*Note:* Test: Wilcoxon signed‐rank. *Indicates statistically significant.

Abbreviations: DMFT, decayed, missing, and filled teeth; GI, gingival index; PI, plaque index.

### 3.4 Changes in Knowledge, Attitude, and Clinical Indicators in the Intervention Group

In the intervention group, after implementing the educational program, both knowledge and attitude scores increased significantly at the 3‐month follow‐up (*p* < 0.001), indicating a positive effect of the intervention on improving cognitive indicators related to oral health. In terms of clinical indicators, the DMFT value increased significantly over 3 months, similar to the control group (*p* < 0.001), which is consistent with the cumulative nature of this indicator; However, the PI index in the intervention group decreased significantly, and the GI index also showed a slight but significant decrease (Table 3). These findings indicate that the educational intervention was able to improve the plaque and gingivitis status in a short period of 3 months.

### 3.5 Relationship Between Knowledge and Attitude and Clinical Indices

A study of correlations showed that there was a positive and significant relationship between the knowledge score and attitude (*p* < 0.001). Also, a negative and significant correlation was observed between knowledge and DMFT (*p* = 0.038) as well as between knowledge and PI (*p* = 0.046), meaning that with increasing knowledge, the cumulative caries rate and the amount of dental plaque were somewhat lower; while the relationship between knowledge and GI was not statistically significant (*p* = 0.366). Attitude score also showed a significant negative correlation with DMFT (*p* < 0.001), but its association with PI and GI was not significant (Table 4). This pattern suggests that cognitive components (knowledge and attitude) are most associated with the cumulative DMFT index and to some extent with plaque amount, and do not necessarily have a direct and strong relationship with the gingivitis index in the short term.

**Table 4:** Relationship between knowledge and attitude with oral health indices at the beginning of the study.

Variable		Attitude	DMFT	PI	GI
Knowledge	Spearman correlation coefficient	0.372	−0.147	−0.141	−0.064
	*p* value	< 0.001*	0.038*	0.046*	0.366
Attitude	Spearman correlation coefficient	—	−0.277	−0.107	−0.069
	*p* value	**—**	< 0.001*	0.133	0.334

*Note:* Test: Spearman correlation coefficient. *Indicates statistically significant.

Abbreviations: DMFT, decayed, missing, and filled teeth; GI, gingival index; PI, plaque index.

### 3.6 Education Level

Distribution of education level in the control group, 28% had a below diploma, 45% a diploma and 27% had a university degree and in the intervention group, 40%, 38% and 22% were reported, respectively (Table 2).

In the analysis of the relationship between education level and the main variables, no significant difference was observed in the knowledge score between different levels of education (low‐level diploma, diploma, and university), and PI and GI indices did not show a statistically significant difference according to education (*p* > 0.05). In contrast, women with university education had significantly higher attitude scores than the diploma and low‐level diploma groups (*p* = 0.003) and the mean DMFT in this group was significantly lower than the other two groups (*p* < 0.001). This indicates that education level, although not necessarily making a clear difference in knowledge and short‐term indicators of plaque and gingivitis, was associated with a more positive attitude and less experience of cumulative caries and could be considered as an important socioeconomic factor in the oral health pattern of pregnant women.

## 4 Discussion

In this study on pregnant women, the implementation of an oral health education program based on face‐to‐face sessions and brochures was able to significantly improve the participants' knowledge and attitude towards oral health and at the same time led to a significant reduction in the plaque index (PI) and a slight but significant improvement in the gingival index (GI) in the intervention group. In contrast, in the control group during the 3‐month follow‐up, knowledge and attitude did not change statistically significantly, and the gingival status and DMFT index showed a worsening trend. According to the predefined score classification (Table 1), the average knowledge score in the intervention group at the baseline was in the “Good” range, but after the intervention it significantly approached the higher values of this range, and the attitude score, which was in the “Positive” level at the beginning, remained in the same category after the training but moved towards the higher end of this range.

In the control group, both indicators practically remained in the same initial classification ranges. As for the DMFT index, as would be expected for a cumulative and irreversible index, only a slight but significant increase was recorded over 3 months; in such a short period of time, the realistic goal is to slow down the rate of increase in DMFT. Furthermore, DMFT in this study was calculated based on clinical examination only and without the use of radiographs, so early and interdental caries were not detected and the true value of DMFT in the study population is higher than the reported figures.

In the present study, the educational intervention resulted in a significant improvement in knowledge and attitude scores towards oral health in the intervention group; this is consistent with a substantial part of the domestic and international literature. For example, in the study by Rezaei et al. on pregnant women in Mashhad, oral health education combined with a quarterly assessment resulted in a significant increase in knowledge and attitude and a reduction in plaque index, although the reduction in gingival index did not reach a significant level and no change was observed in the control group (Rezaei et al. 2023). The results of Mohammadkhah et al. also showed that an educational intervention based on the theory of planned behavior, with several structured training sessions, led to improvements in cognitive constructs, oral self‐care behaviors, as well as DMFT and plaque indices in pregnant women (Mohammadkhah et al. 2023). Similarly, Nickbin et al. reported a significant improvement in behavioral performance related to oral care and a reduction in plaque index in an intervention based on the Health Belief Model (HBM) in pregnant women, which emphasizes the role of structured educational interventions in this population (Nickbin‐Poshtamsary et al. 2018).

Internationally, recent trials, such as the study by Chen et al. in China, have shown that HBM‐based oral health management programs during pregnancy can improve self‐efficacy, health behaviors, and periodontal indices (including PI and GI) in the short term (Chen et al. 2025). Overall, the results of this study, taken together with the above evidence, indicate that even relatively simple and short‐term interventions, if purposefully designed, can improve the cognitive status and, to some extent, the oral clinical status of pregnant women within a few months. However, the differences in the intensity and persistence of effects across studies indicate the importance of the type of theoretical framework, the duration and intensity of training, and the cultural‐organizational context in which the intervention is implemented.

Regarding clinical indicators, the findings of this study showed that in the control group, GI increased significantly over 3 months, while the PI remained almost unchanged; in contrast, in the intervention group, both PI and GI decreased significantly. This pattern is consistent with current knowledge about “pregnancy gingivitis,” as recent reviews and reports indicate that due to hormonal changes and an exaggerated inflammatory response to plaque, between 60% and 70% of pregnant women experience some degree of gingivitis (Sachelarie et al. 2024; Hartnett et al. 2016).

In this context, the finding of increasing GI in the control group of the present study, without a clear change in PI, can be considered a reflection of this increased sensitivity of the gingival tissue during pregnancy. In the intervention group, the significant reduction in plaque may have partially inhibited the aggravating effect of pregnancy on gingivitis and resulted in a relative improvement in GI. This result is consistent with the results of other intervention studies on pregnant women; for example, Nickbin et al. reported that a simple educational program resulted in a significant reduction in plaque index and improved caregiving performance in pregnant mothers (Nickbin‐Poshtamsary et al. 2018), and recent trials based on behavioral models or oral health management programs have also shown a significant reduction in PI and improvement in periodontal indices in the intervention group compared to the control (Liu et al. 2020; Chen et al. 2025). Overall, the results of this study confirm that structured educational interventions can modulate the natural course of gingivitis exacerbation during pregnancy in short periods of time, although the magnitude and sustainability of this effect largely depend on the type of intervention, the duration of follow‐up, and the underlying conditions of the target population.

The findings of this study regarding the role of cognitive variables and education are also noteworthy. A positive and significant relationship was observed between the knowledge and attitude scores, and both indices showed a negative correlation with DMFT, whereas their associations with GI and, in most cases, with PI were weak or not statistically significant. This pattern suggests that higher knowledge and attitude are probably most associated with a reduction in the cumulative caries burden (DMFT) and to some extent with plaque control in the longer term, but in the short term they are not able to explain all the inflammatory changes in the gingiva alone; a finding that is consistent with the known role of hormonal and inflammatory factors in pregnancy gingivitis (Seyyedi et al. 2023; Tenenbaum and Azogui‐Levy 2023; Abubakar et al. 2025).

On the other hand, educational level in this study was not significantly associated with the knowledge, PI and GI scores, but women with university education had a more positive attitude and a significantly lower DMFT than women with a diploma or less education. This result is consistent with KAP (Knowledge, Attitude, and Practices) studies in Iran, which have shown that knowledge, attitude, and especially oral health performance in pregnant women are generally average, while higher education and better socioeconomic status are associated with more favorable attitudes, better care behaviors, and lower values of caries indices (Daneshvar et al. 2023; Tenenbaum and Azogui‐Levy 2023; Abdollahi et al. 2024). In addition to this evidence, the finding of no significant difference in knowledge by education in the present study may reflect the fact that even among educated women, specific knowledge about oral care during pregnancy has not necessarily developed in proportion to general education level; whereas long‐term attitudes and behaviors (including regular dental visits) that affect DMFT are more influenced by socioeconomic factors and service access patterns (Frey‐Furtado et al. 2025; Kamalabadi et al. 2025).

Considering the overall findings, it can be said that this short‐term educational intervention, although simple and inexpensive, was able to partially modulate the natural progression of periodontal problems during pregnancy and to improve pregnant women's knowledge and attitudes regarding oral health.

The short‐term follow‐up, single‐center, and allocation based on the day of visit highlight the need for multicenter trials with longer follow‐up, the use of more complete diagnostic methods, and the design of multi‐session interventions based on behavioral models. However, the evidence from this study on the role of education and cognitive components in oral health‐related behaviors and DMFT index highlights the importance of systematically integrating oral health education into routine prenatal care, especially with special attention to underserved groups. Therefore, it is recommended that health care facilities providing prenatal care focus more on raising knowledge among pregnant women, and in particular on improving their attitudes towards general health and oral health during pregnancy. Educational content should also emphasize the importance of maintaining daily oral hygiene and encourage regular dental check‐ups and dental follow‐up throughout pregnancy as part of comprehensive prenatal care. This recommendation aligns with evidence emphasizing the value of routine oral examinations and periodontal assessment as part of prenatal care (Capasso et al. 2016).

### 4.1 Limitations

A key limitation is the nonrandomized allocation based on day of visit, which may introduce selection bias and limit generalizability. Future studies should use randomized designs to better control for confounding.

## 5 Conclusion

This study showed that adding a structured oral health education program to routine prenatal care significantly improved pregnant women's knowledge and attitudes and reduced plaque and gingivitis indices within 3 months, while in the absence of intervention, gingival status and cumulative caries index showed a more unfavorable trend. Given the cumulative and irreversible nature of DMFT, slowing the rate of its increase, alongside improvements in PI and GI, may be considered a clinically meaningful outcome during pregnancy. Accordingly, integrating targeted oral health education into prenatal care programs—especially for women with lower educational levels—could be a practical strategy to promote maternal oral health during this critical period and reduce the future burden of oral diseases.

## Author Contributions

**Seyyed Amir Seyyedi:** conceptualization (equal), investigation (equal), data collection (equal), writing – review and editing (equal), final approval. **Zohreh Dalirsani:** conceptualization(equal), data curation (equal), methodology (equal), writing – review and editing (equal), final approval. **Fatemeh Bahadori:** conceptualization (equal), data entry (equal), writing – review and editing (equal), final approval. **Nava Askari:** conceptualization (equal), data collection (equal), writing – review and editing (equal), final approval. **Mohadese Maleki:** data interpretation (equal), data collection (equal), writing – review and editing (equal), final approval. **Mohammad Reza Ghorbani Afkhami:** conceptualization (equal), literature review (equal), methodology (equal), data analysis (equal), writing – review and editing (equal), final approval.

## Funding

The authors have nothing to report.

## Conflicts of Interest

The authors declare no conflicts of interest.

## Data Availability

Data of current study are available from the corresponding author on reasonable request. However, for privacy reasons, no individual data allowing identification of participants can be provided.
